# Inflammatory Bowel Diseases: Does One Histological Score Fit All?

**DOI:** 10.3390/diagnostics13122112

**Published:** 2023-06-19

**Authors:** Vincenzo Villanacci, Rachele Del Sordo, Tommaso Lorenzo Parigi, Giuseppe Leoncini, Gabrio Bassotti

**Affiliations:** 1 Institute of Pathology, ASST-Spedali Civili University of Brescia, 25123 Brescia, Italy; villanac@alice.it; 2 Department of Medicine and Surgery, Section of Anatomic Pathology and Histology, Medical School, University of Perugia, 06132 Perugia, Italy; 3 Division of Immunology, Trasplantation and Infectious Disease, Università Vita Salute San Raffaele, 20132 Milan, Italy; parigi.tommaso@hsr.it; 4 1 st Pathology Division, Department of Pathology and Laboratory Medicine, Fondazione IRCCS Istituto Nazionale dei Tumori, 20133 Milan, Italy; giuseppe.leoncini@istitutotumori.mi.it; 5 Gastroenterology and Hepatology Section, Department of Medicine and Surgery, University of Perugia, 06156 Perugia, Italy; gabrio.bassotti@unipg.it

**Keywords:** ulcerative colitis, Crohn’s disease, scoring system, histological mucosal healing

## Abstract

Mucosal healing (MH) is the main treatment target in ulcerative colitis (UC) and Crohn’s disease, and it is defined by the combination of complete endoscopic and histologic remission. The complete resolution of mucosal inflammation should be confirmed by histology but its assessment is not always univocal. Neutrophil infiltration represents the unique histological marker in discriminating the active vs. quiescent phases of the disease, together with crypt injuries (cryptitis and crypt abscesses), erosions, and ulcerations. On the contrary, basal plasmacytosis is not indicative of activity or the remission of inflammatory bowel diseases (IBDs) but instead represents a diagnostic clue, mostly at the onset. Several histological scoring systems have been developed to assess grade severity, particularly for UC. However, most are complex and/or subjective. The aim of this review was to summarize available scores, their characteristics and limitations, and to present the advantages of a simplified mucosa healing scheme (SHMHS) based on neutrophils and their distribution in the gut mucosa. Finally, we overview future developments including artificial intelligence models for standardization of disease assessments and novel molecular markers of inflammation with potential application in diagnostic practice.

## 1. Introduction

The assessment of disease activity is the cornerstone of the modern management of inflammatory bowel diseases (IBDs), thereby shaping its clinical management. Endoscopy represents the gold standard in assessing IBD activities, especially in ulcerative colitis (UC). In the past few decades, remission has been considered as being achieved by obtaining symptomatic relief. However, residual foci of subtle inflammation were found to remain during histology in a subset of cases, thereby raising the bar for treatment goals and mucosal healing (MH) [1]. MH is usually defined by a combination of complete endoscopic and histological remissions. Several scoring systems have been introduced to grade the inflammation in the gut mucosa [2,3]. The Mayo endoscopic score represents the most suitable tool for grading mucosal injuries at endoscopy, and it defines remission as the disappearance of visible inflammation or ulceration (Mayo Score 0 to 1) [4,5]. Endoscopic remission has been found to be associated with a better long-term clinical outcome, including the reduced need for glucocorticoid administration, a lower rate of relapse, hospitalization, and surgical resection [6,7,8]. The occurrence of IBD-associated colorectal neoplasia was also found to be reduced in long-lasting remission [9]. However, endoscopic remission does not equate to histological remission since 14–40% of patients have shown histological evidence of mucosal active inflammation, even though the endoscopic findings were unremarkable [10,11,12]. Early reports by Truelove et al. [13] showed that 37% of patients with normal sigmoidoscopy displayed persistent active inflammation during histology. Thus, the therapeutic target is currently focused on the achievement of histological MH. The histological evidence of active inflammation is a stronger predictor of relapse in UC, less responsiveness to first-line treatment (e.g., NSAIDs and Mesalazine), and hospitalization [1,10]. In remitting UC at endoscopy, the histological evidence of active inflammation was found to be associated with an increased relapse rate [14], as well as an increased risk of developing colorectal neoplasia [15]. According to this evidence, the Food and Drug Administration of the US Department of Health and Human Services recommended that clinical trials should be conducted using both histological assessments and the endoscopic evaluation of UC activity [16]. The role of histology as a predictor of treatment response is less clear in Crohn’s disease (CD) since both the transmural involvement and patchy distribution of the disease limit the role of the mucosal biopsy in assessing the MH [17]. Nonetheless, the histological MH was found to correlate to a lower relapse rate and glucocorticoid administration in remitting CD ileitis, whereas the endoscopic MH did not [18].

## 2. Histopathological Features of IBD

UC usually affects the colorectum and it is characterized by a continuous distribution of inflammation and mucosal injuries. CD can potentially affect all of the gastrointestinal (GI) tracts, from the anus to the mouth, with a discontinuous pattern of inflammation. The terminal ileum is the most common site of onset, followed by the colon, whereas the upper GI location is rare.

The diagnosis of IBD is based on the identification of specific histological features [19]. Among them, the histological hallmarks of UC are represented by the increased density in inflammatory infiltration in the lamina propria, including plasma cells and eosinophils. In particular, an increase in plasma cells is most commonly located at the base of the crypts or in the lower third of the inter-cryptic space in the lamina propria. Such a feature, known as basal plasmacytosis, is of critical diagnostic importance in UC [20]. The number of eosinophils can vary, although they are usually intermingled with plasma cells and their presence (more than three at the base and lateral part of crypts) makes a first diagnosis of UC more likely [21]. Interestingly, an increased number of eosinophils (>60/10 HPF) in treatment-naïve UC patients has been found to correlate with poor treatment responses [22,23].

The presence of neutrophils in the lamina propria marks the active phase of the disease and can lead to glandular and surface epithelial injuries, such as cryptitis, cryptic abscesses, mucosal erosions, and ulcers.

Another histological hallmark is represented by architectural abnormalities, including crypt distortion, namely convoluted, dilated, and non-parallel crypts, crypt branching, which is characterized by dichotomic sprouting in the lower third of the crypt, and crypt atrophy, which is qualified by the crypts being shorter than normal. Architectural abnormalities in the lamina propria have been found in 57–100% of UC cases, and directly correlate to long-standing disease. Conversely, the crypt architecture can be almost normal at onset [20].

Reactive epithelial changes, such as mucin depletion and left-sided Paneth cell metaplasia, can be frequently seen in active and long-standing diseases, respectively. The so-called backwash ileitis is characterized by reactive villous atrophy and inflammation of the more distal part of the ileum. It was found to characterize 20% of patients suffering from active ulcerative pancolitis. A mild degree of neutrophilic infiltration can also be seen in the lamina propria with histology, including cryptitis and crypt abscesses [24]. Moreover, the segmental (incomplete) response to previous treatments has been found to lead to a discontinuous distribution of mucosal inflammation (e.g., the so-called cecal patch) in long-standing diseases [25].

The architectural abnormalities are usually mild and discontinuous in CD, and the mucosal inflammation is patchy when compared to UC. Neutrophil infiltration and epithelial injuries mark the active phase of the disease, as seen in UC. A peculiar feature in CD onset is the infiltration of the superficial epithelium by neutrophils and eosinophils [26,27]. The active inflammation could also manifest as aphthoid ulcers, occurring as mucosal ulceration onto the top of a mucosa-associated lymphoid aggregate, mostly in ileal CD. The two histological features can also be associated with the same mucosal samples. The epithelioid cell granulomas, with or without multinucleated giant cells, are more frequently observed in children than in adults. Caution should be paid in excluding the occurrence of cryptologic granulomas. Reactive epithelial changes could also be detected in ileal mucosa, including irregular villous architecture. Pyloric gland metaplasia has been found in 2–27% of ileal biopsies in CD patients. The detection of the histological features, which indicates that the intestinal inflammation is chronic, represents a crucial tool in distinguishing IBD from acute self-limiting colitis at the onset [19].

## 3. Histological Mucosal Healing

Endoscopic remission has been historically considered a unique parameter in describing MH; however, growing evidence suggests that the endoscopic features should not be considered as an exhaustive descriptor of MH in IBD. Particularly, a residual amount of neutrophil infiltration in the lamina propria was found in more than 40% of patients displaying endoscopic features, which would have pointed toward clinical remission [10,11,12]. This finding has significant implications in clinical practices since histological inflammation was proven to be associated with worse clinical outcomes [1]. Hence, histological evaluations were recommended to be included in defining MH [28]. Histological remission and MH have so far not been completely defined, either in UC or in CD, and disagreement still remains regarding the topic. Firstly, terminology should be properly reassessed, as histological healing and remission are not synonyms, thus, they should not be used as interchangeable terms. Histological remission is defined by a spectrum of features in UC, ranging from the evidence of residual foci in chronic inflammation and architectural distortion to almost normal mucosa [10]. The International Organization of Inflammatory Bowel Disease (IO-IBD) [17] has recently recommended that histological parameters are included as diagnostic treatment-aiding tools in both UC and CD. They included:

1. The disappearance of neutrophil infiltration in the lamina propria;
2. reduction of plasma cell infiltration (to normal values) and disappearance of basal plasmacytosis;
3. reduction of eosinophil infiltration (to normal values).

The presence of basal plasma cells still remains a matter of concern in the histological definition of MH in IBD. Basal plasma cells represent an important diagnostic clue in IBD with a high predictive value at the onset of diseases [20,29,30]. It is commonly used in discriminating IBD from non-IBD colitis [31] and it represents an independent predictor of relapse in clinically remitting UC patients [32]. Accordingly, it has been proposed that basal plasmacytosis should be absent in healed mucosa [33].

Thus, the reappearance of basal plasmacytosis in treated patients could be interpreted as a predictor of forthcoming relapse, even though they are clinically remitting [14].

Furthermore, some studies did not confirm the predictive role of basal plasmacytosis in clinical relapse in UC patients with endoscopic remission [34]. Overall, plasma cells mark the presence of IBD, yet they do not provide any definition of its activity. Their predicting role in a forthcoming relapse in treated patients has been also postulated.

Eosinophils represent a common finding in mucosal samples from IBD patients. The significance of eosinophils as a histological marker of activity is unclear [35]. They have been found to be more numerous than normal in active and quiescent diseases, being usually intermingled with plasma cells both in UC and CD [21,31]. Eosinophil density has been found to correlate to both disease extent and lesser responsiveness to glucocorticoid therapy, although not with the activity of the disease [23,36].

Consistently, the European Crohn’s and Colitis Organization (ECCO) has stated that “eosinophils alone should not be used as a marker of histological activity“ in UC [37].

According to our experience, neutrophil infiltration in the lamina propria, and neutrophil-related injuries in crypts and surface epithelium represent the histological hallmark of disease activity in IBD. Hence, the finding of neutrophil infiltration should be used in distinguishing the active vs. quiescent disease phases, such as in the stomach [38] and it should be considered in the histological MH definition [39]. Accordingly, the absence of both neutrophils and neutrophil-mediated mucosal injuries in the lamina propria was claimed to represent a mandatory requirement for remission to be defined histologically in IBD [37,40]. A notable issue to be addressed remains how to properly approach the histopathological assessment of disease activity in IBD. Firstly, mucosal samples should be evaluated in order to differentiate IBD from non-IBD colitis, and then, the disease activity should be considered. Moreover, thorough clinical-endoscopic data should be available. A proper methodological approach also includes technical requirements, encompassing both the completeness of the mucosal sampling at endoscopy and the proper handling after samples were collected. Particularly, at least two pinch biopsies from both the terminal ileum and the five colorectal segments (cecum-ascending, transverse, descending, sigmoid, and rectum) should be collected. Mucosal ulcers detected by endoscopy should be accurately sampled, including the edges of the lesion [37,40]. The orientation of the mucosal samples is another mandatory requirement to achieve a histological diagnosis [19,37]. Each sample should be sequentially placed by the endoscopist in a straight line onto a cellulose acetate substrate, with the luminal surface upwards [19]. A “clarinet beak-shaped cut” is used to mark the proximal end of the strip in order to provide clues on the segmental location of each sample. Once fixed (37% formaldehyde solution), specimens are processed, and then, paraffin-embedded. A 90 degrees rotation of the filter-specimens complex must be performed by the technicians in order to warrant the optimal trans-sectional orientation of the biopsies. Sequential sectioning is advisable, mostly when the presence of the histological hallmarks is focal or not straightforward [19]. Alternatively, mucosal sampling should use a vial per segment [37].

## 4. Histological Scores in IBD

Several histological scoring systems, approximately 30 for UC and 13 for CD have been proposed to date, both to differentiate the active vs. quiescent disease and to evaluate the therapeutic efficacy. However, they were exceedingly heterogeneous in terminology, histological features, and classification criteria. Moreover, they are tricky, time-consuming, and subjective, which is not suitable for routine diagnostic practices [41,42]. A few of them are currently being used for research purposes, although not in clinical practice since they were burdened by a low interobserver reproducibility both among general and GI pathologists [37,43]. After the first attempt by Truelove et al. [13], the Simplified Geboes Score (SGS), the Robarts Histological Index (RHI), and the Nancy Histological Index (NHI), are the most commonly used for scoring the disease activity in UC. Among them, only the RHI and NHI scores are fully validated and recommended by the European Crohn’s and Colitis Organization (ECCO) for randomized case–control studies, observational studies, and clinical trials. [37]. In fact, NHI is simple and suitable for clinical practice [44]. Currently, there are no validated scoring systems to be applied to CD. Limitations are represented by the patchy distribution of the disease and the transmural extension of the inflammation. The ECCO recommendations stated that GS, RHI, and NHI can be used for intestinal biopsies from CD patients [40], even though they were shown to be insufficient in predicting the clinical outcomes in CD [45]. A simplified score was recently proposed for routine diagnostic practices [46]. It was suitable for the assessment of the histological features, mostly for the disease activity and the MH, in both UC and CD [46].

### 4.1. Geboes Score

It was developed by Geboes et al. in 2000 [47] and has been the most used score in clinical trials, although it is limited by its complexity. It evaluates six histological features (grades), including architectural changes (grade 0), chronic inflammatory infiltrate (grade 1), lamina propria neutrophils and eosinophils (grade 2A and grade 2B, respectively), neutrophils in epithelium (grade 3), crypt destruction (grade 4), and erosions or ulcerations (grade 5). Each grade of the score is further divided into 4 subgrades, ranging from 0 to 3, with the exception of the surface epithelial injury, which ranges from 0 to 4. The subgrades were evaluated using the most inflamed area of the biopsy and without average evaluations. GS can be variably used, either by applying a single score, which ranges from 0 to 6, or by focusing on the most inflamed area in the biopsy and summing the subgrades recorded from all the grades to obtain a total score that ranges from 0 to 22 (known as continuous GS). To reduce its complexity, a simplified GS (SGS) was proposed in 2016 [48] (Table 1).

### 4.2. Nancy Histological Index

The NHI was developed in 2015 [49] and comprises three histological items that define five parameters of the histological activity, including the absence of a significant histological disease (grade 0), chronic inflammation (grade 1), mildly-active disease (grade 2), moderately-active disease (grade 3), and severely-active disease (grade 4). The NHI is characterized by a stepwise evaluation, based on the worst feature observed in the biopsy, to determine the final score. Erosions and ulcerations are categorized in the NHI as grade 4. An active inflammation without erosions or ulceration is graded as grades 2 or 3, according to the severity of the inflammation. Chronic inflammation is defined as grade 1, whereas grade 0 describes the absence of chronic inflammation or if any, as mild (Table 1). The histological remission was defined as NHI = 0 and the histological response as NHI ≤ 1. According to the authors of NHI, the index system is simple and easy to use with good intraobserver and interobserver reproducibility. Furthermore, the orientation of the biopsy specimen is not essential for the assessment of the histological features [49]. Magro et al. [44] demonstrated that NHI and continuous GS strongly correlate to both histological remission and treatment response in UC.

### 4.3. Robarts Histological Index

The RHI was developed in 2017 through a multiple linear regression model [50]. It is based on the GS and includes the four original items that showed the highest inter-rater and intrarater reliability. The items evaluated by RHI included chronic inflammation, neutrophil infiltration, intraepithelial neutrophils, and surface injuries, namely, erosion and ulcerations (Table 1). The final score is obtained by combining the sub-scores from the four items, ranked from 0 to 3, to yield a total score ranging from 0 (absence of disease activity) to 33 (high disease activity). In UC, the histological remission is defined as RHI ≤ 3 (with the sub-scores equal to 0 for both lamina propria and intraepithelial neutrophils, and without erosions or ulceration), while the histological response is defined as RHI ≤ 9 (with sub-scores equal to 0 for intraepithelial neutrophils and without erosions or ulceration) [37]. RHI was recently shown to be strongly correlated with continuous GS in UC [51].

## 5. Simplified Histological Mucosal Healing Scheme (SHMHS)

**Figure 1:**
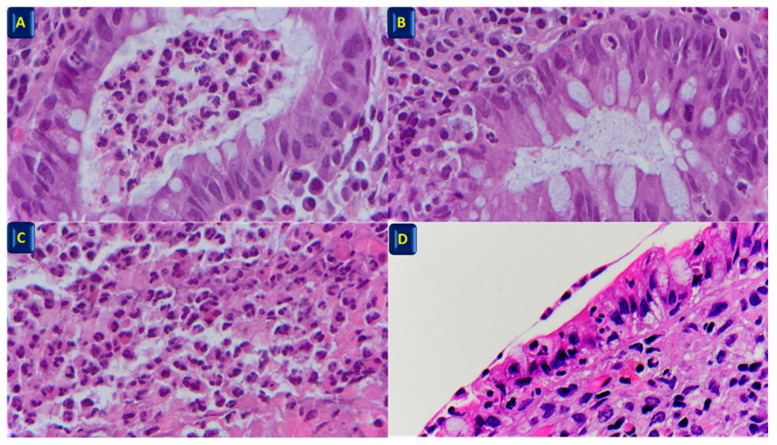
(**A**) Crypt abscess (hematoxylin–eosin dye). Magnification × 40; (**B**) Cryptitis (hematoxylin–eosin dye). Magnification × 40; (**C**) neutrophil infiltration in the lamina propria, intermingled with eosinophils (hematoxylin–eosin dye). Magnification × 40; (**D**) neutrophil infiltration in the surface epithelium (hematoxylin–eosin dye). Magnification × 40.

A simplified scheme was proposed in 2017 [46]. It focuses on neutrophil infiltration as the hallmark of active inflammation, and it can be applied to assess the grade of the activity and MH in both UC and CD. It is composed of eight questions on the histological parameters, including the number of pinch biopsies and the sites of active and quiescent inflammation. The scheme also encompasses three histologic variables, including neutrophil infiltration, cryptitis or crypt abscesses, and epithelial neutrophil-mediated injuries, which encompasses erosions and ulcerations (Figure 1). The SHMHS is subdivided into two parts, both of which include eight questions (Table 1). The first part inquires about the histological features. A score of 1 means that it is present, and it is assigned when at least 1 mucosal fragment has shown that feature during histology; a score of 0 means absent. The second part addresses how mucosal inflammation is distributed in the gut. A question per intestinal segment is proposed, referring to the right colon, the transverse and descending colon, the sigmoid colon, and the rectum. The mucosal sampling from the terminal ileum is only considered in CD patients. The score is assigned as 1 for active inflammation, while 0 is assigned to quiescent disease or normal (non-IBD) mucosa. The disease is considered active when at least one mucosal fragment per segment harbors neutrophil infiltration or neutrophil-mediated epithelial injuries. The disease is considered as quiescent when at least one mucosal fragment per segment shows the histological features of chronic inflammation, including crypt distortion or basal plasmacytosis, and, importantly, when active inflammation is lacking. The scoring system can provide a total score ranging from 0 to 8. A total score ≥ 2 equates to an active disease. The SHMHS has been developed by considering easy-to-find histological features, resulting in a simple and time-sparing tool for assessing disease activity and mucosal healing in IBDs. Such a scheme can be suitably employed in routine clinical practices both by general and GI pathologists because it is simple enough to be applied and less subjective than other schemes. It was shown to be a reliable grading system, achieving similar results when compared to previously validated schemes, such as GS, NHI, and RHI [46]. The score has shown a very good inter-rater agreement (k = 0.94). Moreover, a strong correlation between the Mayo Clinic Endoscopic Score and Simple Endoscopic Score was highlighted in a nationwide multicenter study on both UC and CD [52]. On a similar note, another notable attempt to simplify UC scoring was the PI-CaSSO Histological Remission Index (PHRI) [53]. The score was designed to reduce all the assessments to the sole detection of neutrophils. The mucosa is divided into four functional areas, superficial epithelium, cryptal epithelium, lamina propria, and cryptal lumen. The PHRI ranges from 0 to 4, with 0 considered as remission and any score above that as an active disease. Scores are counted summing the number of areas with at least one neutrophil at 40× magnification. In addition, evidence of erosions or ulcers is considered pathognomonic of inflammation regardless of neutrophils. This approach demonstrated a very good interobserver variability, and correlation with endoscopy at no expense of outcome stratification.

## 6. Future Directions

MH is an evolving concept and so are the tools to assess it. Recently, a wave of innovation due to the development of artificial intelligence (AI) models that are able to automate, expedite, and standardize histological assessments has reached different areas of histopathology [54,55]. Some initial promising AI tools have also been developed in the field of IBDs to grade the activity of inflammation.

The main obstacle in the automation of IBD histological assessment had been integrating the numerous features present in the biopsy and relevant to the overall assessment. In other words, the more complex the evaluation, the more challenging the automation. This has been overcome, as reported above, by simplifying the assessment by considering only the presence or absence of neutrophils [53]. This, then, provided the medical rationale to train a convolutional neural network to detect and count the neutrophils present in small areas of a biopsy and determine whether UC is in remission or activity. When tested in a large dataset of digitalized biopsies, the readings of the machine were accurate compared to the human gold standard, and importantly, stratified the risk of future flares during the follow-up. Furthermore, the remission assessment based on neutrophils largely overlaps with more sophisticated scores, such as NHI or RHI, ultimately, yielding similar results [53,56,57]. In parallel, other groups have developed similar models reaching similar diagnostic performances, all in excess of 80% accuracy [58] and contributing to the enthusiasm on the topic.

So far, such tools have been developed only for UC grading and are not available for CD or the initial IBD diagnosis; however, given the pace of progress in the field, it is reasonable that soon more accurate and versatile tools will become available. These technical innovations will eventually provide a standard assessment of disease activity, which to date remains highly subjective, and hence, burdened by interobserver variability. At the same time, similarly to what is already happening with computer-aided diagnostic systems for polyps detection in endoscopy, these tools will help clinicians in their judgment reducing misdiagnosis, and oversights, and providing precious support for less experienced physicians.

Another promising area of research is novel putative markers of inflammation in IBDs. The treatment options are currently based on immunosuppression leading to disease-transient symptomatic relief. A better understanding of the underlying mechanisms involved in gut-immune homeostasis could help identify new molecular targets that can be employed as therapeutic targets. The glucocorticoid-induced leucine zipper (GILZ) has been found to be involved in mediating some of the glucocorticoid effects, mostly in immune cells. However, GILZ was recently established to exert a secretory role in the gut, being delivered by goblet cells into the lumen. Both in UC and CD, it resulted in an impaired active disease, while it was restored in quiescent disease, thus, correlating with neutrophil infiltration and epithelial injury [59]. Moreover, it was also found to be directly related to MUC2, one of the major components of the mucus barrier, as well as to TLR2 and TLR4, which are involved in several gut mucosal functions, including permeability [60]. Recently, GILZ has gained attention in IBDs as it negatively modulates neutrophil activation, thereby promoting a negative control in the main cellular effector of disease activity in IBD [61,62]. It is worth noting that the exogenous administration of GILZ (TAT-GILZ) was shown to ameliorate the colitis symptoms in a mouse model of IBD, which provides a preclinical assessment of its efficacy in treating colitis [63]. Taken together, these findings suggest GILZ as a molecule of interest in IBD treatments.

## 7. Conclusions

The assessment of histological MH has now become a tool of critical importance in evaluating the treatment efficacy of IBDs. A reliable histological score that can be used to assess the treatment response is still lacking. The available scores are complex and subjective and are mostly limited to research purposes, as they are based on different histological parameters and are not standardized to mucosal sampling at endoscopy. Conversely, the SHMHS could help assess histological remissions during routine diagnostic practices.

## Figures and Tables

**Table 1:** Main scores.

Simplified Geboes Score		Nancy Score		Robarts Score		SHMHS	
Grade 0: No inflammatory activity	0.0: No abnormalities 0.1: Presence of architectural changes 0.2: Presence of architectural changes and chronic mononuclear infiltrate	Chronic inflammatory infiltrate (quantity of lymphocytes and plasmacytes in the biopsy)	0: No increase 1: Mild but unequivocal increase 2: Moderate increase 3: Marked increase	Chronic inflammatory infiltrate	0: No increase 1: Mild but unequivocal increase 2: Moderate increase 3: Marked increase	**Part I. Features**	
						Neutrophils in lamina propria	1: Present 0: Absent
						Cryptitis or crypt abscesses (presence of neutrophils)	1: Present 0: Absent
Grade 1: Basal plasma cells	1.0: No increase 1.1: Mild increase 1.2: Marked increase	Neutrophils in the epithelium	0: None 1: <50% crypt involved 2: >50% crypt involved	Lamina propria neutrophils	0: None 1: Mild but unequivocal increase 2: Moderate increase 3: Marked increase	Erosions or ulcerations (presence of granulation tissue)	1: Present 0: Absent
Grade 2A: Eosinophils in lamina propria	2A.0: No increase 2A.1: Mild increase 2A.2: Marked increase	Ulceration (visible epithelial injury, regeneration, fibrin, and tissue granulation)	0: Absent 1: Present	Neutrophils in the epithelium	0: None 1: < 5% crypts involved 2: < 50% crypts involved 3: > 50% crypts involved	**Part II. Site of involvement**	
						Ileum (CD patient only)	1: Active 0: Quiescent 0: Not involved
Grade 2B: Neutrophils in lamina propria	2B.0: No increase 2B.1: Mild increase 2B.2: Marked increase	Acute inflammatory cell infiltrate	0: None 1: Mild 2: Moderate 3: Severe	Erosion or ulceration	0: No erosion, ulceration, or granulation of tissue 1: Recovering epithelium + adjacent inflammation 1: Probable erosion-focally stripped 2: Unequivocal erosion 3: Ulcer or granulation tissue	Right colon	1: Active 0: Quiescent 0: Not involved
Grade 3: Neutrophils in epithelium	3.0: None 3.1: < 50% crypts involved 3.2: > 50% crypts involved	Mucin depletion	0: None 1: Mild 2: Moderate 3: Severe			Transverse colon	1: Active 0: Quiescent 0: Not involved
Grade 4: Epithelial injury (in crypt and surface epithelium)	4.0: None 4.1: Marked attenuation 4.2: Probable crypt destruction: probable erosions 4.3: Unequivocal crypt destruction: unequivocal erosions 4.4: Ulcer or granulation tissue	Neutrophils in lamina propria	0: None 1: Mild 2: Moderate 3: Severe			Descending Colon	1: Active 0: Quiescent 0: Not involved
						Sigmoid colon and rectum	1: Active 0: Quiescent 0: Not involved
		Basal plasmacytosis	0: None 1: Mild 2: Moderate 3: Severe				
		Serrated architectural (defined as the presence of dilated crypts showing a scalloped lumen)	0: None 1: <5% crypt involved 2: <50% crypt involved 3: >50% crypt involved				

SHMHS: Simplified Histologic Mucosal Healing Scheme.

## Data Availability

No new data were created or analyzed in this study.
